# Genomic heterogeneity of *Dichelobacter nodosus* within and between UK sheep flocks and between age groups within a flock

**DOI:** 10.1186/s12866-020-01769-9

**Published:** 2020-05-01

**Authors:** P. L. Davies, A. M. Blanchard, C. E. Staley, N. J. Bollard, T. J. Coffey, S. Tötemeyer

**Affiliations:** 1 Department of Epidemiology and Population Health, University of Liverpool, Leahurst Campus, Neston, UK; 2 University of Nottingham, School of Veterinary Medicine and Science, Sutton Bonington, UK

**Keywords:** D. nodosus, Footrot, Interdigital dermatitis, Lameness, Sheep, MLST, Serogroup, Molecular epidemiology

## Abstract

**Background:**

Footrot and interdigital dermatitis are endemic infectious diseases in all sheep farming regions, impairing welfare and production. The development of efficacious vaccines against the primary causative pathogen has been hampered by the extensive antigenic diversity of *Dichelobacter nodosus*. Understanding the heterogeneity of the pathogen within and between flocks is essential if the feasibility of bespoke vaccine production is to be assessed for use in the U.K.

**Results:**

In this study 56 ewe and lamb isolates from 9 flocks were compared by *D. nodosus* serogroup and Multi Locus Sequence Type which provides significantly enhanced discriminatory power for molecular epidemiology. Serogroup heterogeneity between flocks ranged from two to five unique serogroups per flock. Three flocks contained isolates of two serogroups, two flocks contained isolates of three serogroups and one flock included isolates of five serogroups. Analysis of 25 isolates from one flock with high prevalence of lameness, identified that serogroup and sequence type was significantly correlated with age. Significantly higher proportion of lambs were infected with serogroup B (principally ST85) as opposed to serogroup H (principally ST86), which predominated amongst adult sheep.

**Conclusions:**

Genomic heterogeneity of the pathogen was significantly lower within flock compared to heterogenicity observed between flocks. Furthermore, this study indicates that within a flock, the host-pathogen dynamics and susceptibility to particular *D. nodosus* strains may be age dependent.

## Background

Footrot is one of the most important infectious diseases affecting the welfare and productivity of sheep globally. Understanding the population structure of the pathogens involved, particularly *Dichelobacter nodosus (D. nodosus)* is critical to the development of efficacious vaccines for footrot control. Vaccines based upon a broad range of *D. nodosus* serogroups, (*Footvax*, MSD) have been shown to provide moderate protection to new infections and reduction in clinical disease severity [9]. The limited efficacy observed has been attributed to antigenic competition [11, 15] and prompted the development of ‘bespoke’ vaccines specific to the serogroups present on individual flocks in Australia with greater success [8]. Understanding the population structure of the pathogen at flock level helps us to understand the challenges and opportunities for the implementation of similar flock-specific vaccination in the UK disease context. Serogrouping (Serogroups A-I and M) has historically been used for classification of *D. nodosus* and has demonstrated significant variation between countries and between flocks [4, 5, 14]. However, genomic techniques such as multi locus sequence typing (MLST) or core genome multi locus sequence typing (cgMLST) allow greater discrimination between isolates and can provide more insights into the dynamics of the disease and its transmission within and between flocks, as has been demonstrated previously in molecular epidemiological studies in other species, such as bovine mastitis using MLST [7, 18]. The aim of this study was to compare the heterogeneity and population structure of *D. nodosus* using discriminatory methodologies, serogroup, cgMLST and MLST sequence type, within and between flocks in the UK.

## Results

Serogroups and MLST classification was performed on 56 isolates from nine flocks of the ten sampled flocks were analysed (Table 1). No isolates could be cultured from the remaining flock. In total six serogroups and 26 unique MLST sequence types were identified (Table 2), while two sequence types (ST88 & ST91) were represented across two different serogroups while the remaining sequence types were serogroup specific (Table 3). Up to seven separate MLST sequence types were identified per flock (range 1–7).

**Table 1:** Serogroup diversity by farm. The number of isolates of each *D. nodosus* serogroup isolated from each of the 9 study flocks from which isolates were successfully cultured. No isolates could be cultured from the tenth flock

Farm id	Serogroup						Total
	A	B	C	E	H	I	
A	0	**13**	0	0	**12**	0	25
B	**2**	**3**	**1**	**2**	0	3	11
C	0	**4**	0	0	**1**	0	5
D	**1**	0	**2**	0	0	0	3
E	0	**1**	**1**	0	**2**	0	4
F	**1**	**2**	0	0	0	**1**	4
G	0	0	0	0	0	**1**	1
H	0	**1**	0	0	0	0	1
I	**1**	**1**	0	0	0	0	2
**Total number of farms**	4 of 9	7 of 9	3 of 9	1 of 9	3 of 9	3 of 9

**Table 2:** MLST sequence type diversity by flock. Numbers of ST's identified per farm

Sequence Type	Farm id								
	A	B	C	D	E	F	G	H	I
**75**	0	**1**	0	0	0	0	0	0	0
**76**	0	0	0	0	0	0	0	0	**1**
**78**	0	**2**	0	0	0	0	0	0	0
**79**	0	**3**	0	0	0	0	0	0	0
**80**	0	**2**	0	0	0	0	0	0	0
**81**	0	**1**	0	0	0	0	0	0	0
**82**	0	**1**	0	0	0	0	0	0	0
**83**	0	0	0	**1**	0	0	0	0	0
**84**	0	0	0	**2**	0	0	0	0	0
**85**	**11**	0	0	0	**1**	0	0	0	0
**86**	**9**	0	0	0	**2**	0	0	0	0
**87**	**2**	0	0	0	0	0	0	0	0
**88**	0	**1**	0	0	**1**	0	0	0	0
**91**	0	0	**3**	0	0	0	0	0	0
**98**	0	0	**1**	0	0	0	0	0	0
**99**	0	0	0	0	0	**1**	0	0	0
**103**	0	0	0	0	0	0	**1**	0	0
**104**	0	0	0	0	0	**1**	0	0	0
**105**	0	0	0	0	0	0	0	**1**	0
**106**	0	0	0	0	0	0	0	0	**1**
**107**	0	0	**1**	0	0	0	0	0	0
**108**	**1**	0	0	0	0	0	0	0	0
**109**	0	0	0	0	0	**1**	0	0	0
**110**	0	0	0	0	0	**1**	0	0	0
**111**	**1**	0	0	0	0	0	0	0	0
**112**	**1**	0	0	0	0	0	0	0	0
**Total ST’s per farm**	6	7	3	2	3	4	1	1	2

**Table 3:** MLST sequence type diversity by serogroup. Numbers of ST's identified per serogroup

MLST Sequence Type	Serogroup					
	A	B	C	E	H	I
**75**	0	**1**	0	0	0	0
**76**	**1**	0	0	0	0	0
**78**	**2**	0	0	0	0	0
**79**	0	0	0	0	0	**3**
**80**	0	0	0	**2**	0	0
**81**	0	**1**	0	0	0	0
**82**	0	0	**1**	0	0	0
**83**	0	0	**1**	0	0	0
**84**	**1**	0	**1**	0	0	0
**85**	0	**12**	0	0	0	0
**86**	0	0	0	0	**11**	0
**87**	0	0	0	0	**2**	0
**88**	0	**1**	**1**	0	0	0
**91**	0	**2**	0	0	**1**	0
**98**	0	**1**	0	0	0	0
**99**	0	**1**	0	0	0	0
**103**	0	0	0	0	0	**1**
**104**	0	0	0	0	0	**1**
**105**	0	**1**	0	0	0	0
**106**	0	**1**	0	0	0	0
**107**	0	**1**	0	0	0	0
**108**	0	**1**	0	0	0	0
**109**	0	**1**	0	0	0	0
**110**	**1**	0	0	0	0	0
**111**	0	**1**	0	0	0	0
**112**	0	0	0	0	**1**	0
**Total**	5	25	4	2	15	5

Isolates of *D. nodosus* were identified from 36 ewes and nine lambs with seven ewes and four lambs generated two isolates from different feet. Only five of the seven ewes produced identical MLST sequence types. Greater diversity was observed in the lambs where two of the four carried multiple sequence types.

**Fig. 1:**
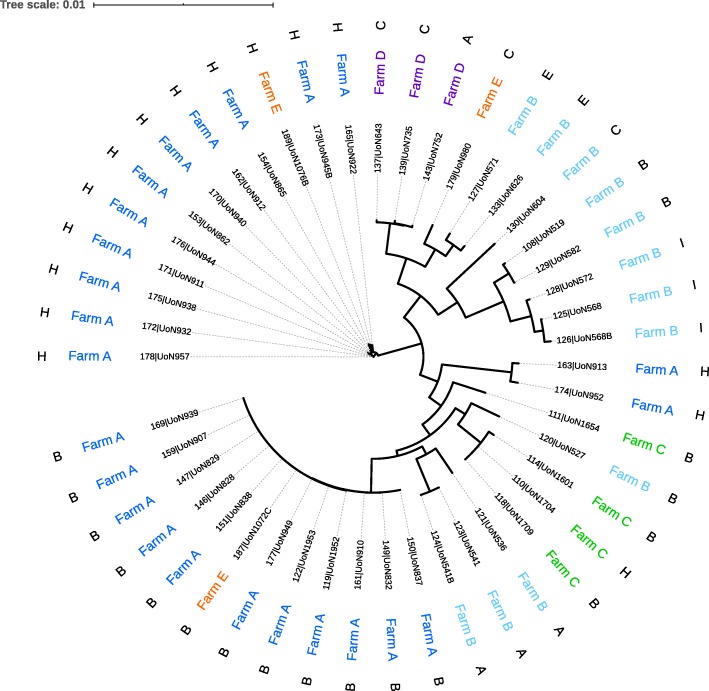
Core genome MLST of *D. nodosus*. Phylogeny inferred using maximum-likelihood double precision, implemented in FastTree Labels from leaf tips outwards are Isolate ID and name, sheep identification number, farm identifier, Sequence type and Serogroup

Genetic heterogeneity within and between flocks was assessed by comparison of cgMLST distance matrix distributions from flocks with at least four isolates from at least three individuals (Fig. 1). The inter-flock heterogeneity was significantly greater compared to that observed within any single flock (*p* < 0.0001) with median distance matrix values of 542 (IQR: 484, 586) and 491 (IQR: 5, 537) respectively. At the level of the individual flock, three of the five flocks carried *D. nodosus* sequence types that were significantly less genetically heterogeneous than the distribution of cgMLST distance matrix values for isolates compared between different flocks. Of the two remaining flocks, isolates from one flock were significantly more genetically heterogeneous than those isolates compared between flocks.

### Within flock analysis of MLST and serogroup distribution by age

Serogroup heterogeneity within flocks ranged up to five serogroups per flock (Table 1). In two flocks serogroups B & H were the only two identified. All isolates originating from lambs in Flock A were typed serogroup B (seven isolates from four lambs, six ST85 and one ST111). Within this flock serogroup B was recovered significantly more frequently from lambs compared to adult ewes (*p* = 0.0325) (four of four lambs vs five of 15 ewes). Within the lambs, serogroup B and sequence type ST85 was dominant representing a significantly larger proportion of the sequence types recovered from lambs compared to the adult sheep (*p* = 0.0181). In contrast, in adult ewes serogroup H was the most common and within this serogroup ST86 was the dominant sequence type identified.

**Fig. 2:**
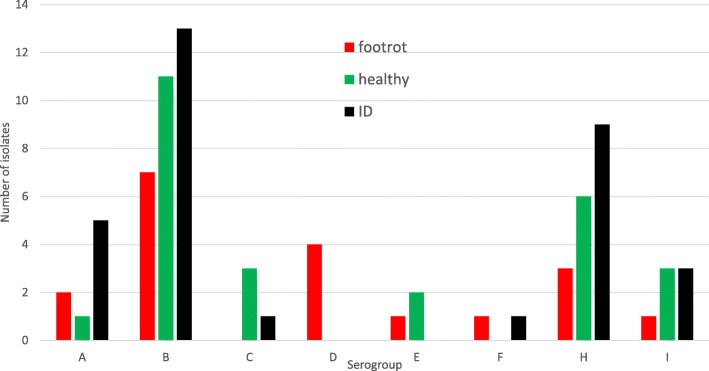
The frequency distribution of isolates by serogroup for each of the three hoof heath categories (Heathy – no abnormalities, Interdigital Dermatitis (ID), Footrot – ID plus underrunning of the hoof capsular horn)

Of the flocks studied, three purchased replacement females, three flocks bred their own female replacements and the remainder practiced a combination of the two policies. Of those flocks purchasing replacements, there were two to six different flocks of origin. Neither the MLST sequence type nor serogroup diversity at the flock level correlated with flock replacement purchasing policies. At the individual ewe level, neither the MLST sequence type nor serogroup diversity correlated with the clinical state of the sampled foot (Fig. 2). All but one isolate (UoN604) was classified as the ‘virulent’ phenotype by aprV2 gene carriage. The isolate diversity determined by sequence type or serogroup did not, in this sample of flocks, correlate with either the flock level prevalence of clinical lameness at the time of sampling or with age, body condition score (1–5), breed, gender, stocking density, management system, husbandry practices specifically related to hoof hygiene or lameness treatment and prevention practices.

## Discussion

Whilst the data presented here represent a small number of lowland flocks and are not intended to provide a definitive description of the *D. nodosus* population structure that would be generalisable to all UK flocks or all farming systems, it does identify a number of interesting and important factors worthy of further investigation. In particular, the limited number of serogroups apparently present on the majority of flocks sampled indicates that bivalent or trivalent vaccines may be appropriate and potentially efficacious in some UK flocks. Serogroups B & H have been used in bivalent vaccines in Australia and may be particularly appropriate for some UK flocks on the basis of the results of this study. However, currently there is no commercially available method of identifying serogroup identity or prevalence in UK flocks and this would need to be addressed if a more sophisticated, targeted approach to *D. nodosus* vaccination is to be attempted.

Whilst serogroups B & H have previously been identified in UK flocks [14], this is the first time that these serogroups and the MLST sequence types ST 85 and ST86 have been shown to be disproportionately associated with specific age groups of sheep (ewes vs lambs). This suggests that host pathogen interactions may change or develop with age causing variation in relative susceptibility to different *D. nodosus* strains. The degree of contact through the use of shared pastures between ewes and lambs may be important factors in determining patterns of colonisation and transmission between these different classes of stock. In this study the ewes and lambs had been co-grazed since the lambs’ birth six - eight weeks prior to the sample collection date so the lambs would have been exposed to all of the strains from the ewes and yet were not equally colonised by them.

Similar to the findings reported by Smith et al. [20], there was no correlation with lesion prevalence or severity (normal, interdigital dermatitis, footrot), additionally, the apparent lack of correlation between the diversity of the *D. nodosus* population and the purchasing policies/biosecurity policies of the flocks may indicate that the bacterial population on the foot changes over time, influenced primarily by the farm environment rather than by the establishment of host populations which are stable over sustained periods. However, larger, multi-flock, longitudinal studies would be required to robustly address these questions with sufficient statistical power to fully elucidate the transmission and colonisation dynamics at the ewe and flock level over time.

The interaction between host and pathogen genetics, environmental conditions and management practices, including antibiotic use and foot bathing, are important to understand the influence on hoof microbiome stability over time. This is outside the scope of the current study. The difficulty in isolating and culturing *D. nodosus* bacteria prior to DNA extraction results in low recovery rate of usable MLST data compared to serogroup testing methods. Improved techniques in bacterial culture and DNA sequencing would substantially improve the quantity of usable data for epidemiological studies into infectious ovine lameness.

## Conclusion

The greater discriminatory power of MLST compared to serogroup (115 sequence types compared to ten serogroups) enhances our ability to understand the transmission of the bacteria between individuals and the wider molecular epidemiology on the hoof and in the pasture or bedding environment. This study demonstrates the diversity of *D. nodosus* strains in commercial flocks and highlights the importance of understanding the transmission dynamics within and between flocks, as well as host – pathogen susceptibility patterns. Both may be influence the development and selection of appropriate control options, such as targeted vaccine strain selection for individual flocks or age groups within flocks.

## Methods

Swab samples (*n* = 2126) were collected from ten sheep flocks situated within Nottinghamshire, Derbyshire and Northamptonshire. All veterinary practices within the east midlands region of England were approached to invite their sheep clients to participate in the research project, of which 15 flocks responded, and the ten largest commercial sheep flocks were selected. All flocks were classified as lowland lamb producers ranging in size from 200 to 1100 ewes. Farmers were requested to present all of their lame ewes on the day of sampling all of which were swabbed (each foot) along with an equal number non-lame sheep selected randomly. The following individual animal data was collected on each animal, age by teeth eruption, body condition score (1–5), breed, gender, hoof heath status (Healthy – no abnormality, Interdigital dermatitis (mild to severe inflammation of the interdigital skin), Footrot – inflammation of the interdigital skin with underrunning of the hoof horn) and diagnosis including CODD. Flock level variables were recorded including stocking density, ewe replacement policy (open vs closed flock replacement policy) and number of source flocks for current breeding ewe population, management system, husbandry practices specifically related to hoof hygiene and lameness treatment and prevention practices; Footvax vaccination, footbath protocols, antibiotic treatment protocol.

Within flock A, 50 lambs and ewes from the flock of 264 ewes and 387 lambs were selected at random. The ewes and lambs had been co-grazed on the same pasture since lambing. The lambs age ranged from 6 to 8 weeks old.

### Bacterial isolation

The bacterial isolation, DNA isolation, sequencing and analysis of sequence data in this study are as described previously in [1]. A total of 2126 Interdigital swabs (E-swabs 480 CE, Copan U.S.A.) were taken from ewes and lambs. The swabs were stored in liquid Amies solution at 5 °C overnight prior to being inoculation of hoof Agar plates containing 4% w/v Bacto Eugon agar (BD, U.S.A.), 0.5% w/v Difco Yeast Extract (BD, U.S.A.), 1.5% w/v BBL Beef Extract (BD, U.S.A.), 1% sodium chloride and 6.6% w/v ovine hoof powder [16]. and incubated under anaerobic conditions at 37 °C. Pure colonies were collected from plates in sterile PBS, washed by centrifugation and resuspended in molecular biology grade water (ThermoFisher, UK). Candidate colonies were identified by visual inspection (*n* = 83 isolates), *bacteriodes spp* and contaminated samples were identified by sequencing and eliminated from the analysis. Fifty six isolates remained and were available for further analysis.

### DNA isolation and sequencing

As described by [1], DNA was isolated using the Qiagen Cador Pathogen Mini Kit, following the manufacturers guidelines and eluted in 60 μl of elution buffer. DNA was sent to MicrobesNG (Birmingham University, U.K.), for sequencing using the Illumina MiSeq at 2 × 250 bp [Raw data is available in the Short Read Archive (PRJNA386733)].

### Analysis of sequence data

Sequence reads were assembled using the A5-MiSeq pipelines, [1] and [6]. The raw reads were analysed for overall quality and sequence adaptors using trimmomatic [3]. Errors in reads were corrected using the SGA k-mer based approach (Simpson et al., 2012). The high quality paired and unpaired reads were then assembled using IDBA-UD [17]. SSPACE [2] was used to scaffold and extend the reads before the clipped and corrected reads were realigned using BWA [12]. The scaffolds were then checked for discordant reads indicative of misassembles and scaffolded again using SSPACE [2].

### Genetic determinations

The assembled contig files were used as the input for IPCRESS [19] a part of the exonerate pipeline. In silico serogroup determination was completed using the *fimA* serogroup PCR primers developed by Zhou et al 2001a [21] and Zhou et al 2001b [22] and phenotypic (aprV2/aprB2) determination made use of the PCR primers created by Frosth et al. [10].

Statistical analysis was conducted in *Minitab18* [13] using Mood’s median test for continuous cgMLST distance matrix data comparison of genetic similarity between bacterial isolates between and within flocks. Fisher’s exact tests were used for comparison of the proportions of isolates for within flock analysis.

For sequence type determination and cgMLST analysis the isolate data was uploaded to the *D. nodosus* MLST database (https://pubmlst.org/dnodosus/) [1].

## Data Availability

All sequence data generated for this study is held in the NCBI SRA and EMBL ENA under the accession number PRJNA386733 and scripts used are available at https://github.com/ADAC-UoN/MLST . The MLST data is held at https://pubmlst.org/dnodosus/.
